# Editorial: Schizophrenia: Human and Animal Studies

**DOI:** 10.3389/fnbeh.2016.00076

**Published:** 2016-04-20

**Authors:** Ahmed A. Moustafa, Thomas W. Weickert, Dorota Frydecka

**Affiliations:** ^1^School of Social Sciences and Psychology, Western Sydney UniversitySydney, NSW, Australia; ^2^Marcs Institute for Brain and Behaviour, Western Sydney UniversitySydney, NSW, Australia; ^3^School of Psychiatry, University of New South WalesRandwick, NSW, Australia; ^4^Neuroscience Research AustraliaRandwick, NSW, Australia; ^5^Department and Clinic of Psychiatry, Wroclaw Medical UniversityWroclaw, Poland

**Keywords:** schizophrenia, cognition, positive symptoms, negative symptoms, dopamine

Schizophrenia is a complex disorder with relatively stable prevalence rates estimated at about 0.5–1% (Saha et al., 2005). Schizophrenia is characterized by the occurrence of various psychopathological symptoms that have been described as positive symptoms (delusions and hallucinations), negative symptoms (apathy, lack of motivation, flat affect, poverty of speech, and social withdrawal), disorganized symptoms (disorganized speech and behavior), and cognitive impairment (Moustafa et al., 2016). Traditionally, cognitive impairment was thought to be evident only in elderly deteriorated patients with schizophrenia; however, over the past 25 years, a body of evidence challenged this view and showed that cognitive dysfunction is a core feature of schizophrenia (O'Carroll, 2000; Weickert et al., 2000; Bora et al., 2010). Cognitive deficits can be moderate to severe across several domains, including attention, working memory, verbal learning and memory, and executive functions (Weickert et al., 2000; Bowie and Harvey, 2006). These deficits pre-date the onset of psychosis and further decline in cognitive functioning is observed in the course of illness in most patients (Trotta et al., 2015). Numerous rehabilitation programs have been developed in order to alleviate the burden associated with cognitive impairment in schizophrenia since cognitive deficits respond poorly to antipsychotic treatment (Tao et al., 2015).

Below we describe articles in this research topic (mostly focusing on cognitive studies in schizophrenia), dividing them into human vs. animal studies.

## Human studies of schizophrenia

Rodriguez et al. investigated the effect of neuropsychological battery on global functioning and quality of life in 36 first psychotic episode of schizophrenia spectrum disorder (FES) patients. The results indicated that patients with FES were significantly impaired in both visuospatial and verbal functioning compared to healthy controls. The authors concluded that the deficit in verbal functioning was a better predictor of global functioning and quality of life than visuospatial functioning.

Chen et al. investigated the nature of prospective memory using event-related brain potentials in 20 symptomatically remitted patients with schizophrenia and 20 healthy controls. The results indicated that patients with schizophrenia performed worse on the prospective memory task compared to the healthy controls. In particular, the prospective positivity amplitude was significantly attenuated in symptomatically remitted schizophrenia patients.

Schott et al. investigated whether dysfunctional processing of novel information by the medial temporal lobe may be a possible neural mechanism that contributes to the pathophysiology of delusions. The authors used fMRI to examine 16 unmedicated patients with paranoid schizophrenia and 20 age-matched healthy controls. Participants completed a visual target detection task that consisted of novel and familiar stimuli, and a standard and a target picture. All of the participants when shown the novel images had activation in the hippocampus, but only healthy controls showed a positive relationship between novelty-related hippocampal activation and recognition memory performance 24 h later, indicating a role of the hippocampus in positive symptoms.

Although previous research (Potvin et al., 2015) suggests that socio-economic status is correlated with cognitive impairments in patients with dementia and schizophrenia, education and income variation may not be the only reasons for such a correlation. Other possible factors include differing life choices, occupations, and environments. The results of Potvin et al. (2015) may lack some experimental control in terms of the dosage and comparison of prescribed antipsychotics. Salem and Moustafa discussed the implications of these findings.

Guell and Bernacer explored the following question: where in the body shall we look for markers of mental disorders? They propose that the constitution of the body's sense organs may be an indicator of mental characteristics. If so, these organs can be affected by mental disorders such as schizophrenia. They suggest the approach of looking for differences in the constitution of the sense organs in order to diagnose mental disorders. Schwitzer et al. provided an insightful commentary on Guell and Bernacer. In their article, they discussed the role played by visual attention in the occurrence of schizophrenia symptoms, focusing on retinal and early visual processing deficits.

Wang et al. investigated the neural correlates of the thalamus and examined whether thalamic resting state networks are different in patients with schizophrenia. The authors used a standard seed-based whole-brain correlation (72 patients with schizophrenia, 73 healthy controls). Patients with schizophrenia had enhanced thalamic connectivity with bilateral precentral gyrus, dorsal medial frontal gyrus, middle occipital gyrus, and lingual gyrus. These patients also had reduced connectivity in bilateral superior frontal gyrus, anterior cingulate cortex, inferior parietal lobe, and cerebellum. The findings indicated that the thalamus is over-connected during resting state in schizophrenia.

Schwab et al. investigated saccadic latency modulation in patients with schizophrenia and their first-degree relatives. Participants (13 patients with ICD-10 schizophrenia, 10 first degree relatives, 24 controls) performed two visual tasks: a color task and a Landolt ring orientation task. Saccadic latency was measured with a video-based oculography. Results indicated that saccadic latencies were not modulated by visual processing of different content for patients with schizophrenia, but saccadic latencies were modulated for relatives and health controls.

Hori et al. investigated the relationship between serum BDNF levels and decision making. They compared patients with schizophrenia (86) to healthy controls (51) in terms of their decision making ability and serum BDNF levels. Scores on the Iowa Gambling Task were lower for patients with schizophrenia compared to the controls. There was a negative correlation between the mean net scores on the final two blocks and serum BDNF levels. The authors concluded that the poor performance of patients with schizophrenia on the Iowa Gambling Task may be related to impaired sensitivity to reward and punishment due to depressive symptoms and reduced serum BDNF levels.

Frydecka et al. compared the level of working memory impairments across patients with schizophrenia (*n* = 23), psychotic bipolar disorder (*n* = 19), non-psychotic bipolar disorder (*n* = 24), and healthy controls (*n* = 18). The results indicated that on more demanding working memory measures the group with schizophrenia and psychotic bipolar disorder performed poorer than the non-psychotic bipolar disorder group and healthy controls. There was a positive correlation of working memory performance with antipsychotic treatment and a negative correlation with depressive symptoms in bipolar disorder and with negative symptoms in the schizophrenia subgroup.

Zhuo et al. investigated changes in resting-state functional connectivity density (rsFCD) in schizophrenia (*n* = 95) and healthy controls (93). The results indicated that patients with schizophrenia had increased rsFCD in the bilateral striatum and hippocampus and decreased rsFCD in the bilateral sensorimotor cortices and right occipital cortex.

Wotruba et al. investigated functional brain correlates in the anticipation and reception of rewards and examined their possible association with symptoms in unmedicated people at risk for psychosis (*n* = 21) and healthy controls (*n* = 24). The results indicated that the same brain areas were recruited during processing of reward information in both at risk individuals and healthy controls. In contrast, during the anticipating rewards stage the at risk individuals showed additional activation in the posterior cingulate cortex and the medio- and superior-frontal gyrus.

Garbarini et al. investigated whether patients with schizophrenia have an altered sense of agency during the observation of others' movements. A circles-lines task was used, in which participants had to draw lines while observing an examiner's hand draw circles. Results indicated an interference effect of the examiner's hand drawing circles on their hand drawing lines.

Moustafa et al. provided an overview of homocysteine metabolism in psychiatric disorders with a focus on cognitive correlates. Several biological mechanisms linking homocysteine to biological underpinnings of psychiatric disorders were discussed and future directions and perspectives were indicated.

## Animal studies of schizophrenia

Below, we summarize animal studies we covered in our research topic.

Falsafi et al. examined which GABAB-containing receptor complexes are involved in a multiple T maze task. Twenty trained and untrained mice were exposed to the multiple T maze task. The results indicated that training increased GABAB1/1A containing receptor complexes in the hippocampus.

Oliveras et al. investigated whether the Roman high-avoidance inbred strain (RHA-I) display prepulse inhibition (PPI) compared to Roman low-avoidance rats (RLA-I) and genetically heterogenous (NIH-HS) rats. More specifically, they examined whether PPI deficits predict spatial working memory impairments. The results indicated that the RHA-I rats had PPI and spatial working memory deficits compared to the other two groups.

Nakao et al. examined the role of beta-anchoring and -regulatory protein (BARP) in higher brain functions. BARP knockout (KO) mice were generated and a comprehensive behavioral test battery was undertaken. The results indicated that these rats showed greatly reduced locomotor activity.

Gruter et al. investigated the cellular mechanisms that may underlie hippocampal dysfunction in psychosis. Male Wistar rats were treated with irreversible NMDAR-antagonist, MK801. After 4 weeks, the treatment led to the loss of hippocampal long-term potentation (LTP). Expression of the early gene (Arc) was enhanced in the hippocampus of controls by spatial learning. The authors concluded that in psychosis, deficits in hippocampus-dependent memory may be due to the loss of hippocampal LTP.

Niu et al. investigated whether the prepulse inhibition (PPI) response can be disturbed by the blockage of olfactory input. The results indicated that the PPI of the startle response was impaired by the blockage of olfactory sensory input. Microinjection of lidocaine/MK801 into the olfactory bulb impaired the PPI. The authors concluded that the olfactory system plays a role in the PPI of the startle response in rats.

Pinnock et al. investigated whether activate muscarinic and potentially nicotine receptors (nAChRs) expressed by pontine receptors are responsible for the nicotine enhancement of prepulse inhibition (PPI). The results indicated that nAChR antagonists had limited effects on PPI. However, PnC microinfusions of the non-α7nAChR preferring antagonist TMPH into the PnC significantly reduced PPI.

DeAngeli et al. investigated whether a change in the concentration of kynurenic acid (KYNA; which is increased in patients with schizophrenia) would affect reward related behavior. The rats that were treated with L-kynurenine showed increased sign-tracking behavior. The authors concluded that exposure to KYNA may increase the salience of cues related to reward. They also examined the effect of exposure to KYNA on hippocampal long-term potentiation (LTP). After a burst of high-frequency stimulation the rats treated with L-kynurenine showed no LTP, whereas vehicle-treated rats showed robust LTP.

Richter et al. translated a human stability-flexibility paradigm to mice. Mice were trained in a touchscreen-paradigm to discriminate visual cues. Results provided evidence for the *Dual State Theory* that assumes an antagonistic relation between cognitive flexibility and stability.

Ding et al. investigated whether *N*-methyl-D-aspartate receptor (NMDR) and Ca^2+^permeable α-amino-3-hydroxy-5-methyl-4-isoxazole-propionic acid receptor (AMPAR) activation in the striatum is necessary for cognitive flexibility. The findings suggested that NMDR-mediated glutamate transmission in the nucleus accumbens contributes more to cognitive execution than the dorsal striatum.

In short, in this topic, we attempted to cover different aspects of schizophrenia symptoms, using both human and animal studies. The articles covered here explained the complexity of behavioral and neural abnormalities in schizophrenia, and further suggest future work in this area.

## Author contributions

All authors listed, have made substantial, direct and intellectual contribution to the work, and approved it for publication.

## Conflict of interest statement

The authors declare that the research was conducted in the absence of any commercial or financial relationships that could be construed as a potential conflict of interest.
